# Differences in Physiological Responses of Two Tomato Genotypes to Combined Waterlogging and Cadmium Stresses

**DOI:** 10.3390/antiox12061205

**Published:** 2023-06-02

**Authors:** Rong Zhou, Lifei Niu, Jian Yin, Fangling Jiang, Yinlei Wang, Tongmin Zhao, Zhen Wu, Weimin Zhu

**Affiliations:** 1 College of Horticulture, Nanjing Agricultural University, Nanjing 210095, China; zhour@njau.edu.cn (R.Z.);; 2 Department of Food Science, Aarhus University, Agro Food Park 48, 8200 Aarhus N, Denmark; 3 Vegetable Institute, Jiangsu Academy of Agriculture Science, Nanjing 210095, China; 4 Shanghai Academy of Agriculture Sciences, Shanghai 201400, China

**Keywords:** tomato, waterlogging, cadmium stress, combined stress, ROS metabolism, plant growth

## Abstract

Waterlogging and heavy mental (e.g., cadmium) stress are two primary threats to crop growth. The combination of abiotic stresses was common and frequent, especially in the field condition. Even though the effects of individual waterlogging and cadmium on tomato plants have been widely investigated, the response of tomatoes under combined waterlogging and cadmium stress remains unclear. This study aimed to clarify and compare physiological, biochemical characteristics and plant growth of two tomato genotypes under individual and combined stress. Two tomato genotypes (‘MIX-002’ and ‘LA4440’) were treated under control, waterlogging, cadmium stress and their combination. The results showed that chloroplast ultrastructure of tomatoes under individual and combined stress was damaged with disordered stroma and grana lamellae. The H_2_O_2_ (hydrogen peroxide) content and O_2_^·−^ (superoxide anion radical) production rate of plants under all the three stresses was not significantly higher than the control except for ‘LA4440’ under the combined stress. Antioxidant enzymes actively responded in the two tomato genotypes, as shown by significant increase in SOD activity from ‘MIX-002’ under waterlogging and combined stress and from ‘LA4440’ under cadmium. Meanwhile, CAT activity of ‘MIX-002’ under waterlogging and ‘LA4440′ under combined stress significantly decreased, and the POD activity of ‘MIX-002’ under combined stress significantly increased as compared with the respective control. The APX activity of ‘MIX-002’ and ‘LA4440’ under combined stress was significantly lower and higher than the respective controls. This indicated that tomato plants were able to secure redox homeostasis and protect plants from oxidative damage through the synergetic regulation of antioxidant enzymes. Plant height and biomass of the two genotypes under individual and combined stress significantly decreased, which could be a direct result from the chloroplast alteration and resource re-allocation. Overall, the effects of combined waterlogging and cadmium stress were not simply the sum of individual effects on two tomato genotypes. Distinct ROS (reactive oxygen species) scavenging systems of two tomato genotypes under stresses suggest a genotype-dependent antioxidant enzymes regulation.

## 1. Introduction

With the increase in frequency and severity of heavy rainfall or flooding, there has been a growing interest in understanding the impact of waterlogging on the growth of plants, especially crops species [1,2,3]. The negative impacts of waterlogging on individual plants are largely characterized as reduced gas exchange, since the diffusion of gases through water was slower than in air [3]. Human activities such as urban disposal and metal manufacturing have led to an increase in cadmium content [4]. Cadmium stress has emerged as another threat to crop production, which posed a concern to public health due to the possibility of cadmium entering the food chain system [5,6]. Cadmium toxicity negatively affects various processes of plants, including seed germination, seedling growth, plant development, crop yield and so on, by damaging important components such as chloroplasts and mitochondrion [4].

Tomato (*Solanum lycopersicum* L.) is one of the most important vegetable crops in the world with 5,167,388 ha harvested area and 366,015 hg/ha production in the year of 2021 (http://faostat.fao.org/, accessed on 20 May 2023), and it is a model plant for studying abiotic stress response as well. Previous research has primarily focused on the response mechanism of tomato to individual stresses, such as waterlogging stress [2,7,8] and cadmium stress [9,10,11]. On one hand, waterlogging has been found to negatively affect vegetative organs as indicated by lower leaf chlorophyll content and decreased photosynthesis [3,12]. The negative impacts on reproductive organs of tomato plants were found as well, as shown by few fruits and lower average weight [8]. On the other hand, even though cadmium stress did not cause decreased leaf chlorophyll content, tomato plants exhibited reductions in plant dry weight concurrently with the increasing CdCl_2_ content [9]. Chu et al. (2020) found that cadmium stress significantly altered the transcriptional level of metal transport-related genes in tomatoes, resulting in the inhibition of iron (Fe) and zinc (Zn) uptake [10]. Cadmium stress led to decreased F_v_/F_m_ (maximum potential quantum efficiency of photosystem II), photosynthetic rate, root growth and vitality as well as biomass accumulation in tomato plants by increasing reactive oxygen species (ROS) accumulation and lipid peroxidation [11].

More importantly, tomatoes are frequently exposed to concurrent abiotic stresses caused by unfavorable environments [12], such as waterlogging and cadmium stress. Provinces, such as Henan, Shandong, Jiangsu and Hainan in China, are the main tomato production areas, where increased frequency and strength of concentrated extreme or abnormal precipitation can cause waterlogging and lead to a serious threat to field-grown tomato plants. Meanwhile, agricultural soil has been contaminated with cadmium due to industrialization, applications of sewage and use of various fertilizers, pesticides and insecticide, making cadmium toxicity an agricultural and environmental issue worldwide [4,6]. Thereby, field-grown tomato plants can suffer combined waterlogging and cadmium stress. A large proportion of tomato plants are grown in greenhouses by applying specific substrates and fertilization involving reutilization of water and nutrition, which implied an increased risk of cadmium accumulation [13]. Together with errors in irrigation, greenhouse-grown tomato plants may be exposed to this combined stress as well.

However, compared with individual stress, investigation of combined effects of waterlogging and cadmium stress on plants such as tomato at the morphological and physiological level is still insufficient. Chloroplasts are one of the most sensitive organelles of plants to abiotic stress such as waterlogging [7]. In plants, ROS can be induced and accumulated when the plants are exposed to abiotic stresses, including waterlogging [14] and cadmium stress [4]. The ROS, such as hydrogen peroxide (H_2_O_2_), superoxide anion radical (O_2_^·−^) and hydroxyl radicals (OH), can cause membrane lipid peroxidation and cell damages [15,16]. Previous reviews have described the role of ROS in plants’ responses and acclimations to stress combination and its role in sensing and mediating rapid systemic signaling [11,15,16]. It has been suggested that the accumulation of proline as an osmolyte can sustain water status and hydraulic conductivity of cucumber during waterlogging conditions [17]. The MDA (malondialdehyde), a final byproduct of membrane lipid peroxidation, was used as a key parameter to evaluate the level of damage caused by stress [18]. Even though the effects of waterlogging and cadmium stress on the above parameters of plants are well established, the physiological and biochemical responses of tomatoes, especially from the perspective of ROS mechanism to combined waterlogging and cadmium, remains unclear.

In this study, we investigated the plant phenotype, chloroplast structure, antioxidant system and biomass accumulation of two different tomato genotypes under waterlogging, cadmium stress and their combination. We aimed to characterize and compare the morphological and physiological responses of the two tomato genotypes under combined waterlogging and cadmium stress and clarify the reason for the different responses. Our hypothesis was that combined waterlogging and cadmium stress posed unique responses in tomato plants, which were not additive effects of individual waterlogging and cadmium stress. This study will provide novel insights into the response of tomato plants to combined waterlogging and cadmium stress.

## 2. Materials and Methods

### 2.1. Plant Materials and Growth Conditions

Tomato genotypes ‘MIX-002’ and ‘LA4440’ from the Laboratory of Vegetable Physiology and Ecology, Nanjing Agricultural University, were used as plant materials. Seeds were sown in 72-hole trays (54 cm length, 28 cm width) with the mixture of peat, perlite and vermiculite (volume ratio 2:1:1). Seedlings were grown in a climate chamber (RGD-1000C, Ningbo, China) with 14-h light (400 µmol m^−^^2^ s^−^^1^, LED light source) at 25 °C and 10-h dark at 18 °C, where the relative humidity was 60 ± 5%. After 21 days of sowing, the plants with three leaves were transferred to pots (6.5 cm height, 6.5 cm diameter) containing the same substrate mixture. The plants were cultivated in the chambers under the same and stable environmental conditions (e.g., light, temperature, etc.) before the treatments.

### 2.2. Experimental Treatments

After 28 days of sowing, the plants with four leaves were randomly divided into four treatments with 30 plants per treatment. The treatments included control (CK), individual waterlogging stress (WL), individual cadmium stress (Cd) and combined waterlogging and cadmium stress (WL + Cd). The WL treatment was performed mainly according to Zhou et al. (2022) [19], where the plants together the small pot (6.5 cm height, 6.5 cm diameter) were put in a big pot (10.8 cm height, 11.1 cm diameter) being filled with water. The water spilled over the soil surface (equal to the height of small pot) for 1 cm, but did not spill over the big pot, which was checked daily to ensure the steady level of waterlogging. As suggested by Ondrasek et al. (2022), Cd–Cl complexes could represent a major form of Cd taken up to plant [20]. Hence, the Cd treatment was achieved by adding 100 mL of 250 mg/L CdCl_2_ solution daily into the pots. The WL+Cd treatment was performed by adding 100 mL of 250 mg/L CdCl_2_ solution followed by adding water until the water was above the soil surface for 1 cm. During the 10 days of the stress treatment, when the Cd treatment were carried out per day, the CK were irrigated with the equal amounts of water (100 mL).

### 2.3. Measurements

We performed the following measurements with three biological replicates from three plants per genotype per treatment.

#### 2.3.1. Chloroplast Ultrastructure and Chlorophyll Fluorescence

On day 8 of the treatments, the 3rd fully expanded leaf from top to bottom of the tomato plant was taken for chloroplast ultrastructure observation and chlorophyll fluorescence measurements. The leaves were collected and cut into small pieces (3 mm length, 2 mm width). Then, the leaves were vacuumed and fixed with 2.5% glutaraldehyde at 4 °C for 8 h. Phosphate buffer was applied to wash the leaves three times with 15 min per time. Then, the leaves were fixed with 1% osmium acid for 1 h and washed again with phosphate buffer. The leaves were sequentially dehydrated with 30%, 50%, 70%, 80% and 90% ethanol for 20 min, respectively. Afterwards, the leaves were dehydrated three times (30 min per time) using 100% ethanol. After dehydration, the leaves were soaked three times (at least 30 min per time) using propanol. The samples were treated with a mixture of encapsulant and acetone for four hours and finally with pure encapsulant overnight. After the above processes, the samples were sliced into 50–90 nm slices using ultratome (LEICA UC7, Wetzlar, Germany). The samples were double stained with uranyl acetate and lead citrate and finally observed; photos were taken using the transmission electron microscope (TEM, Hitachi 7800, Tokyo, Japan).

Tomato plants were adapted to dark conditions for 30 min before the measurements of F_v_/F_m_. Subsequently, we took the leaves and measured the F_v_/F_m_ using the modulated chlorophyll fluorescence imaging system (IMAG-MAXI, HeinzWalz, Effieltrich, Germany). For fluorescence, 450 nm blue light wavelength was applied.

#### 2.3.2. MDA, H_2_O_2_, Proline Content and O_2_^·−^ Production Rate

On day 9 of the treatments, the 3rd fully expanded leaves were prepared for the measurements of MDA content, H_2_O_2_ content, proline content and O_2_^·−^ production rate. The following indices were measured using the enzyme marker (CYTATION 3, BioTek, Winooski, VT, USA).

The determination of MDA content was based on the thiobarbituric acid (TBA) method [21]. The 0.2 g fresh leaves were mixed with 5% TCA solution, and then the samples were ground thoroughly and centrifuged. Afterwards, the supernatant was taken and mixed with an equal volume of 67% TBA solution. The mixture samples were shaken well and boiled in a water bath for 30 min. The cooled rapidly with cold water and then. The 200 μL supernatant was taken in the ELISA plate after centrifuge. The absorbance values of the sample were taken records at 450 nm, 532 nm and 600 nm.

The H_2_O_2_ content was detected using potassium iodide spectrophotometry according to Chakrabarty and Datta [22]. The 0.2 g leaves were ground in liquid nitrogen and added with 0.1% TCA solution. Then, the samples were centrifuged at 3000 rpm (revolutions per minute) for 20 min. The supernatant was added with 1 M KI solution and 100 mM potassium sulfate buffer with dark reaction for 1 h. Finally, the absorbance values of the samples at 390 nm are measured with 0.1% TCA solution as reference.

The proline content was measured based on the ninhydrin colorimetric method [23]. The 0.2 g leaves were mixed with 2 mL 3% sulfosalicylic acid solution. The mixture was ground well, transferred to centrifuge tube and boiled in water bath for 10 min. The samples were centrifuged after cooling and the supernatant was mixed with equal volumes of glacial acetic acid and acidic ninhydrin. Then, the samples were boiled in a water bath for 30 min and mixed with toluene after cooling. Toluene was applied as reference. The upper layer solution was taken to determine the absorbance value at 520 nm.

The production rate of O_2_^·−^ was determined according to Ke et al. (2017) [24]. The phosphatic buffer solution (PBS, 0.05 M, pH 7.8) and 10 mM hydroxylamine hydrochloride solution were added to the enzyme extract. The mixture was at 25 °C for 20 min. Subsequently, sulfanilic acid and α-naphthylamine were added and the mixture was at 25 °C for 20 min. After centrifugation at 3000× *g* rpm for 3 min, the absorbance values of the samples at 530 nm were measured.

#### 2.3.3. Antioxidant Enzyme Activities

On day 9 of the treatments, the 3rd fully expanded leaf was taken to measure antioxidant enzyme activities. The 0.2 g leaf was mixed with PBS (4 °C) and then ground at ice bath temperature. The samples were mixed carefully and centrifuged at 4 °C and 12,000× *g* rpm for 20 min. Supernatant was used to determine the enzyme activities. The activities of superoxide dismutase (SOD), peroxidase (POD), catalase (CAT) and ascorbate peroxidase (APX) was determined based on nitrogen blue tetrazolium method [25], guaiacol method and spectrophotometric method [26,27,28], respectively. For SOD activity, one unit was defined as amount of enzyme to inhibit NBT photochemical reduction by 50%. The units of POD, CAT and APX activities were defined as an enzyme activity unit with OD value changing 0.01 per minute.

#### 2.3.4. Plant Morphology and Biomass Accumulation

On day 10 of the treatments, we measured plant height, stem diameter, fresh and dry weight of shoot and root. Plant height was obtained by measuring vertical distance from cotyledonary node to growing point using a ruler. Stem diameter was investigated by measuring the diameter at 1 cm above the cotyledonary node using vernier caliper. Afterwards, the fresh weight of the shoot was obtained by cutting the plants from cotyledonary node and immediately measuring the weight of above-ground tissue. The root was taken from the substrate and carefully washed to measure the weight as the fresh weight of root. The dry weight of the shoot and root was obtained by drying the fresh samples at 80 °C until constant weight.

### 2.4. Data Analysis

All the measurements included three biological replicates. Data were subjected to statistical analysis of variance (ANOVA) using the SPSS package (SPSS 25.0) at the level of *p* < 0.05. The correlation analysis was conducted using Origin software. Vector diagrams were made using Microsoft Excel 2016.

## 3. Results

### 3.1. Chloroplast Ultrastructure Observation and Chlorophyll Fluorescence Measurements

**Figure 1:**
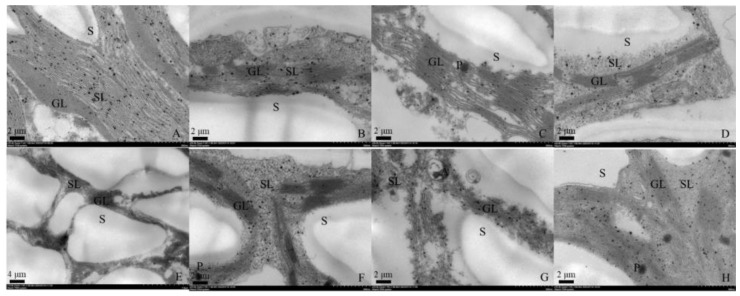
Chloroplast ultrastructure of leaf mesophyll cell from genotype ‘MIX-002’ (**A**–**D**) and ‘LA4440’ (**E**–**H**). (**A**–**D**) indicated the tomato genotype ‘MIX-002’ under control, waterlogging stress, cadmium stress and combined stress, respectively; (**E**–**H**) indicated the tomato genotype ‘LA4440’ under control, waterlogging stress, cadmium stress and combined stress, respectively. GL: grana lamella; SL: stroma lamella; S: starch grain; P: osmiophilic granules.

Both genotypes under control conditions showed normal ellipse-shaped chloroplasts with closely arranged lamellar structure (Figure 1A,E). The chloroplasts of plants under WL treatment exhibited dissolved membrane and damaged lamellar structure with partially disintegrated grainy lamellar (Figure 1B,F). By comparison, Cd stress caused more severe damage on chloroplasts than WL stress. The chloroplasts of two genotypes under WL stress showed disordered, fractured and even disintegrated stroma lamellae, the grana lamellae of which squeezed and deformed (Figure 1C,G). Similar damages to the chloroplast structure were shown in individual and combined stress (Figure 1D,H). Additionally, osmiophilic granules were found in ‘MIX-002’ under Cd stress and in ‘LA4440’ under combined stress (Figure 1C,H).

**Figure 2:**
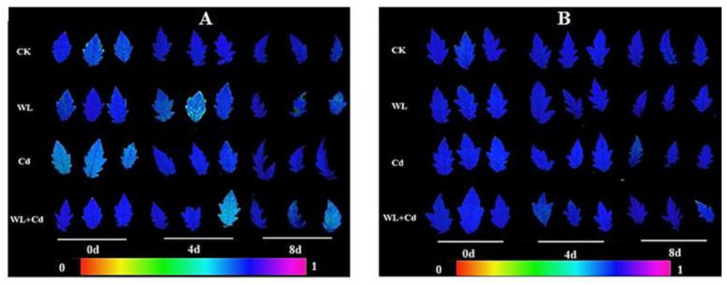
Leaf chlorophyll fluorescence of (**A**) ‘MIX-002’ and (**B**) ‘LA4440’ under CK (control), WL (waterlogging stress), Cd (cadmium stress) and WL + Cd (combined stress). There were three leaflets per genotype per treatment per time point. The color bar indicated the value of maximum potential quantum efficiency of photosystem II (F_v_/F_m_).

The genotype ‘MIX-002’ had a lower F_v_/F_m_ in part leaves under WL on 4 d (1/3) and 8 d (2/3) and WL + Cd on 4 d (1/3) and 8 d (1/3), as shown in Figure 2A by light-blue leaves. The F_v_/F_m_ of ‘LA4440’ generally did not show significant difference, since all the three leaves per treatment on 0 d, 4 d and 8 d exhibited dark-blue leaves (Figure 2B). However, even though the F_v_/F_m_ of some leaves was observed to decrease in ‘MIX-002’ (Figure 2A), the chlorophyll fluorescence of two genotypes were not significantly affected by the current stress treatments (data not shown).

### 3.2. Key Regulators in Antioxidants System

**Figure 3:**
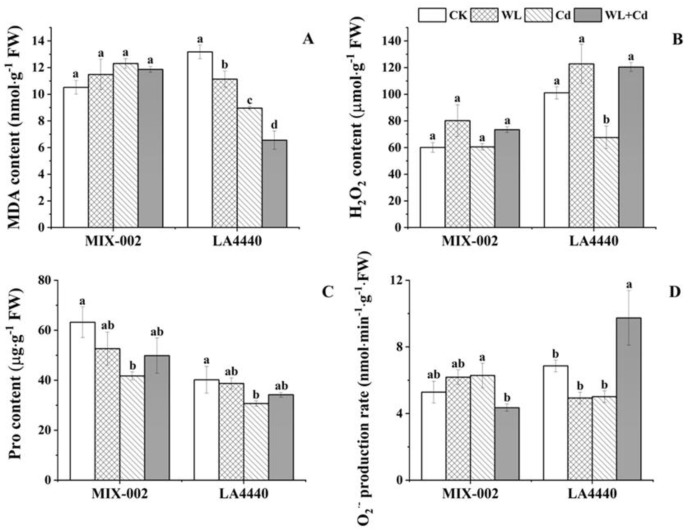
(**A**) MDA (malondialdehyde) content, (**B**) H_2_O_2_ (hydrogen peroxide) content, (**C**) proline/pro content and (**D**) O_2_^·−^ (superoxide anion radical) production rate of two tomato genotypes (‘MIX-002’ and ‘LA4440’) under CK, WL, Cd and WL + Cd. The CK, WL, Cd and WL + Cd indicated control, waterlogging stress, cadmium stress and combined stress, respectively. The bars showed means ± SE (*n* = 3). Different lowercase letters on the bars indicated significant differences within the same genotype (*p* < 0.05).

The MDA and H_2_O_2_ content in leaves of ‘MIX-002’ remained unchanged (Figure 3A,B). The MDA content of ‘LA4440’ significantly decreased under stress conditions with highest decline under combined stress as compared with the control (Figure 3A). The H_2_O_2_ content in leaves of ‘LA4440’ under Cd stress was significantly lower than the control, waterlogging and combined stress (Figure 3B). The proline content in both genotypes under Cd stress significantly decreased, compared to the control (Figure 3C). The O_2_^·−^ production rate of ‘MIX-002’ under combined stress was significantly lower than that under individual Cd stress, while that of ‘LA4440’ under combined stress was significantly higher than the control and individual stresses (Figure 3D).

### 3.3. Antioxidant Enzyme Activities

**Figure 4:**
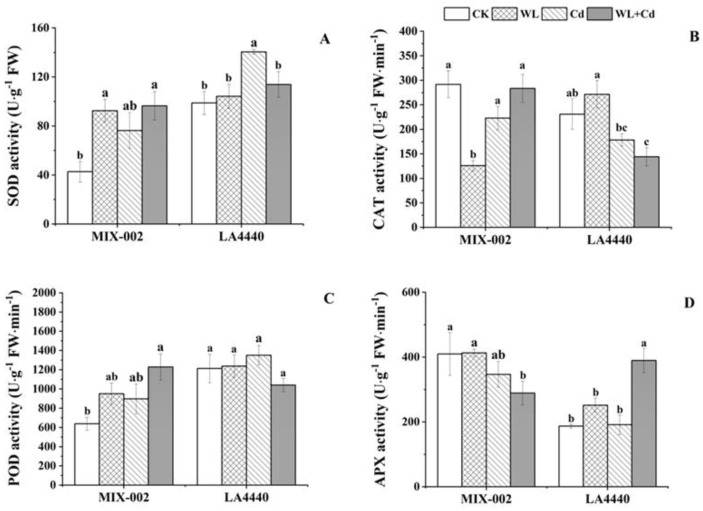
Antioxidant enzyme activities including (**A**) SOD (superoxide dismutase), (**B**) CAT (catalase), (**C**) POD (peroxidase) and (**D**) APX (ascorbate peroxidase) of two tomato genotypes (‘MIX-002’ and ‘LA4440’) under CK, WL, Cd and WL + Cd. The CK, WL, Cd and WL + Cd indicated control, waterlogging stress, cadmium stress and combined stress, respectively. The bars showed means ± SE (*n* = 3). Different lowercase letters on the bars indicated significant differences within the same genotype (*p* < 0.05).

The SOD activity of ‘MIX-002’ significantly increased under WL and combined stress, while that of ‘LA4440’ significantly increased under Cd stress, as compared with respective control (Figure 4A). The CAT activity of ‘MIX-002’ under WL stress and ‘LA4440’ under combined stress was significantly lower than the respective control (Figure 4B). The POD activity increased only in ‘MIX-002’ under combined stress compared to the control (Figure 4C). Furthermore, the APX activity in ‘MIX-002’ was lower under combined stress, while in ‘LA4440’ it was higher under combined stress than their respective control (Figure 4D).

### 3.4. Biomass Accumulation

**Figure 5:**
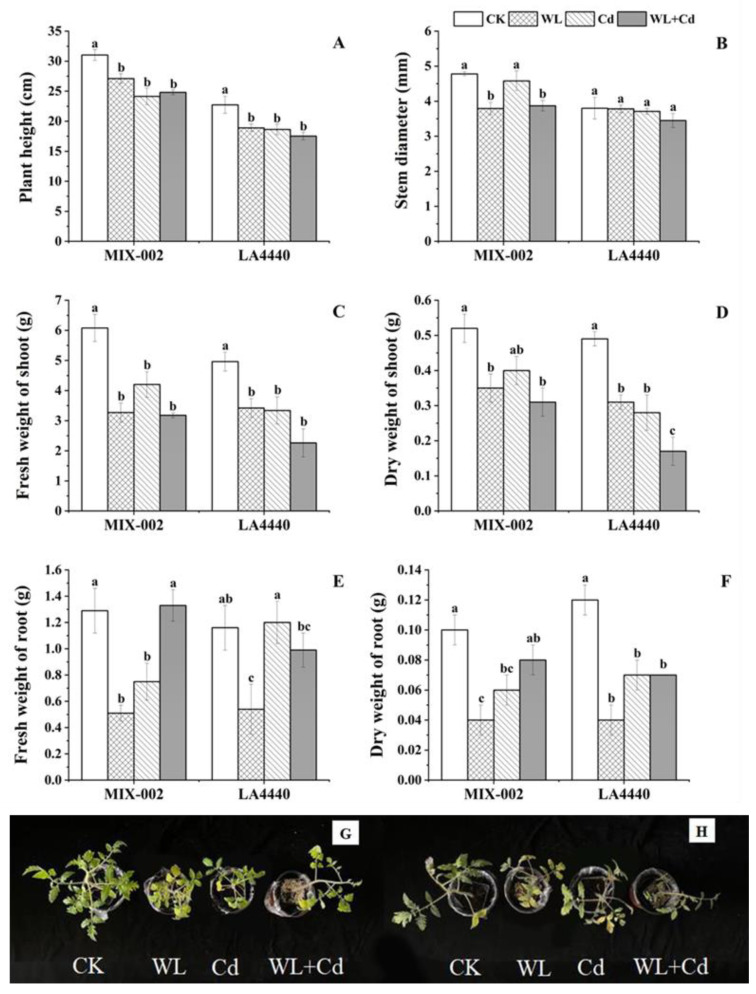
Effects of different stress treatments on plant growth indices of tomato plants. (**A**) Plant height, (**B**) stem diameter, (**C**) fresh weight of shoot, (**D**) dry weight of shoot, (**E**) fresh weight of root, (**F**) dry weight of root, (**G**) phenotype of ‘MIX-002’ and (**H**) phenotype of ‘LA4440’. CK, WL, Cd and WL + Cd indicated control, waterlogging stress, cadmium stress and combined stress, respectively. The bars showed means ± SE (*n* = 3). Different lowercase letters on the bars indicated significant differences within the same genotype (*p* < 0.05).

Under individual and combined stress, both genotypes had significant reductions in plant height, stem diameter, shoot fresh and dry weight compared with the control, except for ‘MIX-002’ under Cd stress and stem diameter of ‘LA4440’ (Figure 5A–D). The individual stresses including WL and Cd significantly decreased the root fresh and dry weight of ‘MIX-002’ (Figure 5E,F). Three stress treatments (WL, Cd, WL + Cd) significantly decreased the root dry weight of ‘LA4440’ (Figure 5F). Plants of both genotypes under all three stress treatments showed smaller plant size with reduced plant height, yellowish leaves and fewer roots than those under the control, corresponding to the plant weight (Figure 5G,H and Figure S1).

### 3.5. Effects of Individual Factors and Their Interactions on the Parameters

**Figure 6:**
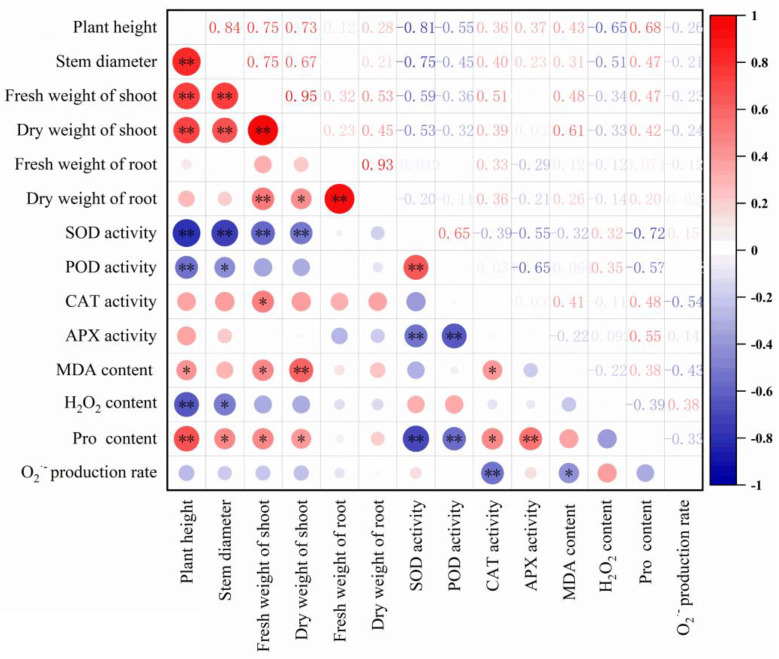
Correlation analysis of the measured parameters. The key 14 parameters were shown in the x and y axis. The numbers in the square indicated the correlation coefficients. ‘*’ and ‘**’ indicated that *p* < 0.05 and *p* < 0.01, respectively.

Here, significant correlations between the parameters were found (Figure 6). Plant height showed significant correlations with stem diameter, fresh and dry weight of shoot, SOD and POD activity, MDA, H_2_O_2_ and proline content. Shoot fresh and dry weight were found to be significantly correlated with root dry weight, SOD activity, MDA and proline content. Additionally, SOD activity was significantly correlated with POD, APX activity and proline content.

The individual factor, cultivar, had significant effects on most measured parameters except for root weight and CAT activity (Table 1). The waterlogging factor significantly affected plant growth indices, MDA content, H_2_O_2_ content and APX activity (Table 1). The heavy mental factor significantly affected plant height, shoot weight, MDA and proline content as well as SOD activity (Table 1).

Cultivar and WL had significant interaction on stem diameter, root fresh weight and MDA content (Table 1). WL and Cd showed significant interaction on plant height and plant biomass (Table 1). The three factors had significantly interactive effects on O_2_^·−^ production rate. Moreover, the interaction between two and three factors had significant effects on enzyme activities (Table 1).

## 4. Discussion

With the prediction of more frequent and heavy rainfall and flooding events, it is crucial to investigate the regulatory mechanism of plants in response to waterlogging in order to maintain a successful agricultural production [3]. Previous research has investigated the combined effects of waterlogging and other abiotic stress, including WL + heat [29], WL+ salt [30], WL+ salt + CO_2_ concentration [19] and WL+ salt + heat [31]. However, the interaction between waterlogging and cadmium stress on tomato plants remained unknown.

### 4.1. Damage of Combined Waterlogging and Cadmium Stress on Tomato Plants Was Not Accumulative

Kołton et al. (2020) concluded that chlorophyll fluorescence can be utilized to identify the sensitivity of tomato to waterlogging stress [7]. Here, F_v_/F_m_ was not an appropriate parameter for the selection and identification of waterlogging tolerant tomatoes since there was no significant difference under control and waterlogging stress. This could be partially explained by different sensitivities of chlorophyll fluorescence parameters. The other reason could be that waterlogging might not directly affect the photosystem II (PSII) but might result in affecting the water splitting site of PSII. In accordance, Kołton et al. (2020) reported that F_v_/F_0_ (ratio of the photochemical and non-photochemical processes in photosystem II or PSII), PI ABS (performance index on an absorption basis), DI0/RC (flux of energy dissipated in processes other than trapping per active PSII reaction center) or Area (area above the OJIP transient and Fm line) were better in selecting tomato sensitivity to waterlogging stress than F_v_/F_m_ [7].

Tomato plants exposed to individual waterlogging and cadmium stress exhibited oxidative damage by inducing the production of excessive reactive oxygen species (ROS) [4,14]. On the contrary, we found that excess water did not give rise to ROS accumulation as indicated by unchanged H_2_O_2_ content and O_2_^·−^ production rate in this case. In previous studies, the H_2_O_2_ and MDA content increased in tomatoes treated by waterlogging for 15 days [14]. Waterlogging for 14 and 28 days induced proline and H_2_O_2_ production, while inhibited MDA content in tomatoes [12]. Furthermore, the H_2_O_2_ content, O_2_·^−^ production rate and MDA content of tomato shoots under individual heat and salt stress and their combination were generally lower than the control [32]. Our study found that only the MDA content of ‘LA4440’ under stress significantly decreased (Figure 3), indicating that the regulatory mechanism of the antioxidant system was dependent on the genotype and stress condition.

**Figure 7:**
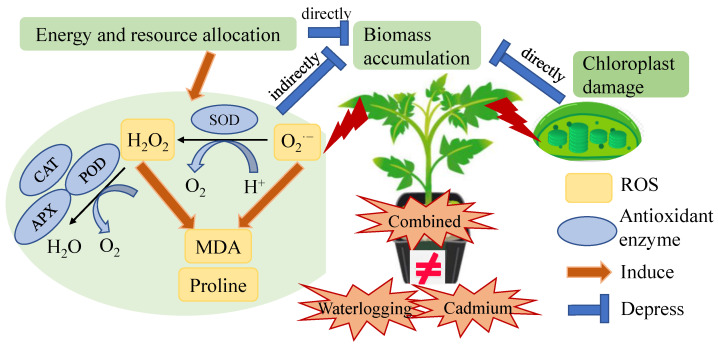
Effects of waterlogging and cadmium on tomato as reflected by antioxidant enzyme and growth indicators. The effects of combined waterlogging and cadmium stress on tomato plants were not equal to the sum of individual stress effects.

In this study, it was observed that the damage caused by the combined waterlogging and cadmium stress on tomato plants did not accumulate as compared with individual stress (Figure 7). This could be attributed to the following three reasons. Firstly, the damage caused to the chloroplasts of plants under combined stress was similar to that caused by individual stress (Figure 1). Secondly, the content of proline and H_2_O_2_ were stable in tomato under combined stress with even low MDA content (Figure 3). Thirdly, the decrease in plant height and biomass accumulation was similar between individual and combined stress (Figure 5). Thereby, co-exposure of tomato plants to waterlogging and cadmium stress did not result in more severe damage in our case, corresponding to our previous report that tomato plants under multiple stress usually exhibited unique responses [33,34]. The potential reason could be that the combined waterlogging and cadmium treatment resulted in the dilution of cadmium in the early stage of the combined stress when the tomato plants can acclimate to the mild cadmium stress. However, previous studies found that the soil water conditions did not affect total Cd in the treelets of *Inga laurina* [35], indicating that the waterlogging might not affect Cd content in tomato plants. By comparison, the bio-concentration factor of cadmium and waterlogging stress was lower, but the translocation factor of cadmium and waterlogging stress was higher than that of cadmium stress in Bermuda grass (*Cynodon dactylon*) [36]. Tomatoes under combined stress can exhibit both shared and unique responses as compared with individual stress [33], which made the combined stress a new and complex state of stress condition to investigate.

### 4.2. Two Tomato Genotypes under Combined Stress Exhibited Different Coordinate Regulation of Antioxidant Enzymes

Within the two genotypes, only for ‘LA4440’ under combined stress did the MDA content decrease but the O_2_^·−^ production rate increase (Figure 3). High levels of ROS cause damage on plants at stress by oxidating important cell components, which activated the antioxidant system, including enzymatic and non-enzymatic components, to remove excess ROS [37]. Here, the H_2_O_2_ and proline content was steady in both genotypes under WL+Cd (Figure 3), indicating there were no redox disorders when the tomato plants were exposed to combined stress. Thereby, antioxidant enzymes successfully maintained the redox homeostasis and protected the leaves from membrane lipid peroxidation and oxidative damage in tomato at stresses, as previously described by Sousa et al. (2022) [32]. However, the two genotypes exhibited distinct changes from the perspective of antioxidant enzymes. The plants of ‘MIX-002’ showed increased SOD and POD activity but decreased APX activity under combined stress, while that of ‘LA4440’ had lower CAT activity but higher APX activity as compared with the respective control (Figure 4). Increased activities of antioxidant enzymes showed an activated defense system in plants being induced by environmental changes [38]. Thereby, we concluded that the synergetic regulation between the antioxidant enzymes played positive roles in protecting tomatoes from oxidative damage caused by combined waterlogging and cadmium stress.

Waterlogging is known to inhibit the development and growth of plants by restraining the aerobic respiration and reducing energy metabolism [3]. Previous studies have shown that shoot weight decreased significantly in tomato under waterlogging for two and four weeks [12]. Similarly, we found that the plant height and biomass accumulation (fresh and dry weight of shoot and root) of tomato treated by waterlogging were significantly lower than the control (Figure 5 and Figure S1). A similar phenomenon of decreased biomass of both aboveground and underground sections in tomato plants under an unfavorable environment was found by Sousa et al. (2022) [32], where the plants were treated at 100 mM NaCl (60 mL per pot) and 42 °C for 4 h per day. Here, oxidative damage caused by ROS accumulation in tomato under stress conditions did not directly cause biomass loss (Figure 7). This was consistent with the findings by Sousa et al. (2022) [32], where the accumulation of antioxidant metabolites and relevant enzymes actively responded in tomatoes under heat and salt stress. The alteration of chloroplast ultrastructure can directly affect photosynthetic apparatus state and photosynthesis capacity [39]. Together with our results, we concluded that the waterlogging and cadmium stress caused the chloroplast damage and induced the re-allocation of energy and resource towards the ROS defense system (antioxidant enzymes), which heavily compromised the plant growth and gave rise to biomass loss (Figure 7).

## 5. Conclusions

In summary, we concluded that the effects of combined waterlogging and cadmium stress on tomato plants were not additive. The synergetic regulation between the antioxidant enzymes of two tomato genotypes under combined stress successfully kept the ROS in normal range. The biomass accumulation of tomato was lower under combined stress than the control. Thereby, excess ROS was not the direct reason why the tomato plants under stress condition failed to accumulate more biomass; instead, the damage to chloroplast structure and function, resulting in decreased photosynthesis capacity and the activation of ROS defense system inducing resource re-allocation, may have contributed to the biomass loss of tomato under waterlogging and cadmium stress. Moreover, the absorption of Cd into tomato plants and how the ROS regulatory mechanism played a role in responding to combined waterlogging and cadmium stress need further clarification.

## Figures and Tables

**Table 1:** Significant levels of the main factors (cultivar, WL, Cd) and their interaction on the physiological parameters.

Index	Main Factors			Interactions			
	Cultivar	WL	Cd	Cultivar × WL	Cultivar × Cd	WL × Cd	Cultivar × WL × Cd
Plant height	**	**	**	ns	ns	*	ns
Stem diameter	**	**	ns	*	ns	ns	ns
Fresh weight of shoot	*	**	**	ns	ns	*	ns
Dry weight of shoot	*	**	**	ns	ns	ns	ns
Fresh weight of root	ns	**	ns	*	ns	**	ns
Dry weight of root	ns	**	ns	ns	ns	**	ns
MDA content	**	*	**	**	**	ns	ns
H_2_O_2_ content	**	**	ns	ns	ns	ns	ns
Proline content	**	ns	*	ns	ns	ns	ns
O_2_^·−^ production rate	*	ns	ns	ns	ns	ns	**
SOD activity	**	ns	**	**	ns	*	ns
CAT activity	ns	ns	ns	ns	**	*	**
POD activity	**	ns	ns	*	ns	ns	ns
APX activity	**	*	ns	**	**	ns	**

Note: WL and Cd indicated individual waterlogging and cadmium stress. Significant correlations are indicated with asterisks (* *p* < 0.05, ** *p* < 0.01, ns: No significant).

## Data Availability

Data is contained within the article.

## Supplementary Materials

The following supporting information can be downloaded at: https://www.mdpi.com/article/10.3390/antiox12061205/s1, Figure S1: Root morphology of two tomato genotypes under CK, WL, Cd and WL + Cd conditions. The CK, WL, Cd and WL + Cd corresponded to control, waterlogging, cadmium and combined stress, respectively.
