# Supplementary material for: Differences in Physiological Responses of Two Tomato Genotypes to Combined Waterlogging and Cadmium Stresses
Source: Antioxidants (Basel). 2023 Jun 2;12(6):1205. doi: 10.3390/antiox12061205 (PMC10295130; doi:10.3390/antiox12061205)
Supplement: Supplementary file 1 [file antioxidants-12-01205-s001.zip › antioxidants-2337479-supplementary.pdf]

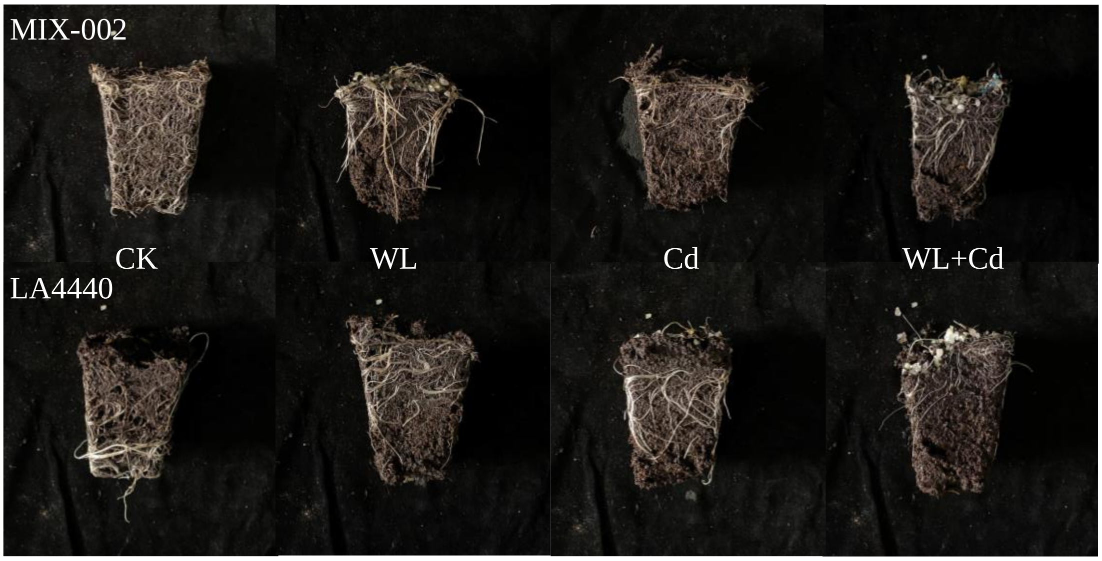

Figure S1. Root morphology of two tomato genotypes under CK, WL, Cd and WL+Cd conditions. The CK, WL, Cd and WL+Cd corresponded to control, waterlogging, cadmium and combined stress, respectively.
